# The Relativistic Proton Spectrometer: A Review of Sensor Performance, Applications, and Science

**DOI:** 10.1007/s11214-023-00962-2

**Published:** 2023-04-05

**Authors:** J. E. Mazur, T. P. O’Brien, M. D. Looper

**Affiliations:** 14745 Lee Road, Chantilly, VA 20151 USA

**Keywords:** Radiation belts, Radiation effects, Trapped protons, Magnetosphere, Solar energetic particles

## Abstract

The Relativistic Proton Spectrometer (RPS) on the Van Allen Probes spacecraft was a particle spectrometer designed to measure the flux, angular distribution, and energy spectrum of protons from $\sim60~\text{MeV}$ to $\sim2000~\text{MeV}$. RPS provided new information about the inner Van Allen belt: a nearby region of space that had been relatively unexplored because of the difficulties of making charged particle measurements there and the associated hazards to satellite operations. We met the primary mission objective of providing accurate data for the AP9 radiation specification model at the high energies where there were little to no data prior to the Van Allen Probes mission. Along the way, we were able to demonstrate the long-term stability of parts of the Inner Belt by comparison with short-lived space science missions that operated decades prior to Van Allen Probes. The most significant surprises were the agreement between RPS and some of those historical measurements and the discovery of a trapped population of $>30~\text{MeV}$ leptons at the outer edge of the inner belt. This end-of-mission paper summarizes the instrument performance, calibration, data products, and specific science and engineering results, and includes suggestions for future investigations of intense radiation fields like those found within the inner belt.

## Introduction and Overview

The inner Van Allen radiation belt is a near-Earth region of space that, despite its proximity, has not been probed and characterized as much as other regions of the Earth’s magnetosphere. While low-Earth orbiting satellites routinely pass through the lowest-altitude extent of the inner belt, those platforms necessarily sample only a small fraction of the trapped particle population that exists mainly above $\sim1500~\text{km}$. As a result, much of the science and applications knowledge that we have about the inner belt relates to a tiny fraction of the particles that actually reside there.

One has to look back to the early days of the 20th century space age to find missions that deliberately transited the most intense portions of the inner belt. Systems like OV120 (1972) and Telstar (1963) that we highlight below provided limited measurements of the trapped protons for both science exploration as well as engineering questions. Orbits at low altitude ($<1000~\text{km}$), medium 12-hour orbit altitude ($\sim20{,}000~\text{km}$), and geostationary altitude ($\sim36{,}000~\text{km}$) became more standard for science and applications beginning in the early 1970s. The harsh radiation environment in the inner belt and the more frequent use of other orbits meant that the inner belt became a no-go zone for space systems, unless the system had to transit the region in order to reach higher altitude.

**Fig. 1:**
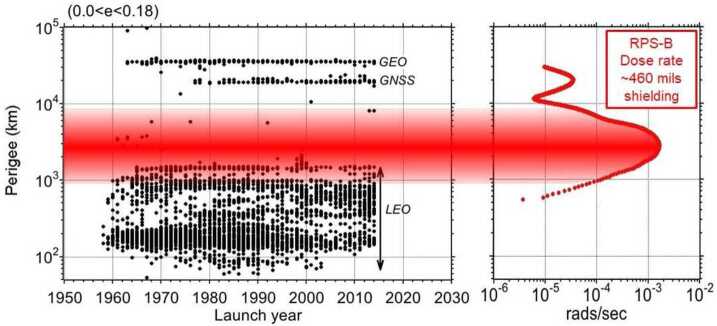
Launch dates and orbital altitudes of satellites with roughly circular orbits (eccentricities between 0.0 and 0.18). Note that in this display, some perigees below $\sim100~\text{km}$ resulted from orbits becoming nearly circular as the satellites approached re-entry. The rightmost figure is an altitude profile of the empirical average dose rate behind thick shielding measured within the RPS-B sensor

To illustrate the avoidance of the inner belt we show in Fig. 1 a compilation of satellite launch dates filtered by vehicles with approximately circular orbits. Some of the lowest-perigees in this analysis resulted from nearly-circular orbits as the satellites approached re-entry. The adjacent plot is representative of the long-term average dose rate behind thick shielding in RPS, described in more detail below. Aside from a few missions in the 1960s, most have avoided orbits that spend a large fraction of their time in the inner belt at higher altitudes where the environment is most stressing.

Our goal with the RPS investigation was to revisit the inner belt, specifically the trapped proton environment from 60–2000 MeV, using the low-inclination orbit of the Van Allen Probes to enable us to sample protons over as wide a range of equatorial pitch angles as possible. We used lessons from prior charged particle spectrometers to design a system that would meet our measurement requirements in an intense and penetrating background of energetic protons. Passive shielding or active anti-coincidence methods that involve surrounding the detectors with one or more vetoing detectors were not viable options; we were targeting protons that could penetrate half a meter of aluminum shielding with a relatively hard energy spectrum.

**Fig. 2:**
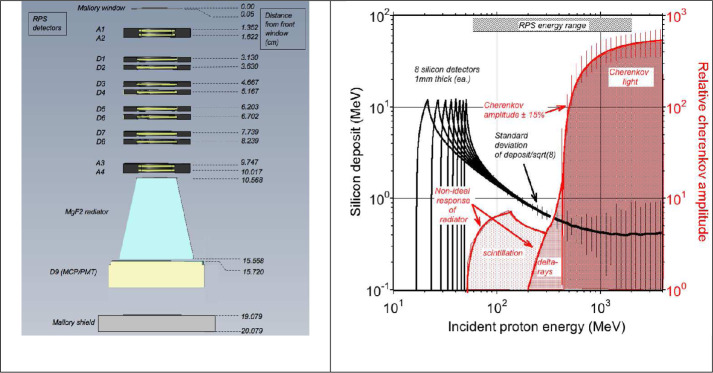
Schematic of the RPS physics package including all active solid-state detectors, the Cherenkov radiator, and microchannel plate/photomultiplier tube (MCP/PMT; leftmost graphic). Protons that triggered all detectors would then be analyzed for a pulse of Cherenkov light above threshold as illustrated on the right. The Cherenkov system proved most useful for distinguishing between forward and backward going protons (down and up, respectively, in the physics package diagram)

Those performance considerations led to the RPS design requiring a 10-fold coincidence in a stack of silicon solid-state detectors for a valid measurement (Mazur et al. 2012). RPS was not a standard range particle telescope. Figure 2 illustrates the measurement principle and the physical design of the sensor including the stack of silicon detectors and the Cherenkov light subsystem. Inner belt protons triggered these detectors from any angle of incidence.

Using solid angles alone and assuming isotropy, the desired forward-going protons that result in 10-fold coincidence events were only about 1% of the total incident proton population. The specific RPS sensor and electronics design resulted in maximum event processing deadtime of $>70\%$ at the peak inner belt intensities near $\text{L}=1.5$. These factors illustrate the challenges of making accurate measurements there.

**Fig. 3:**
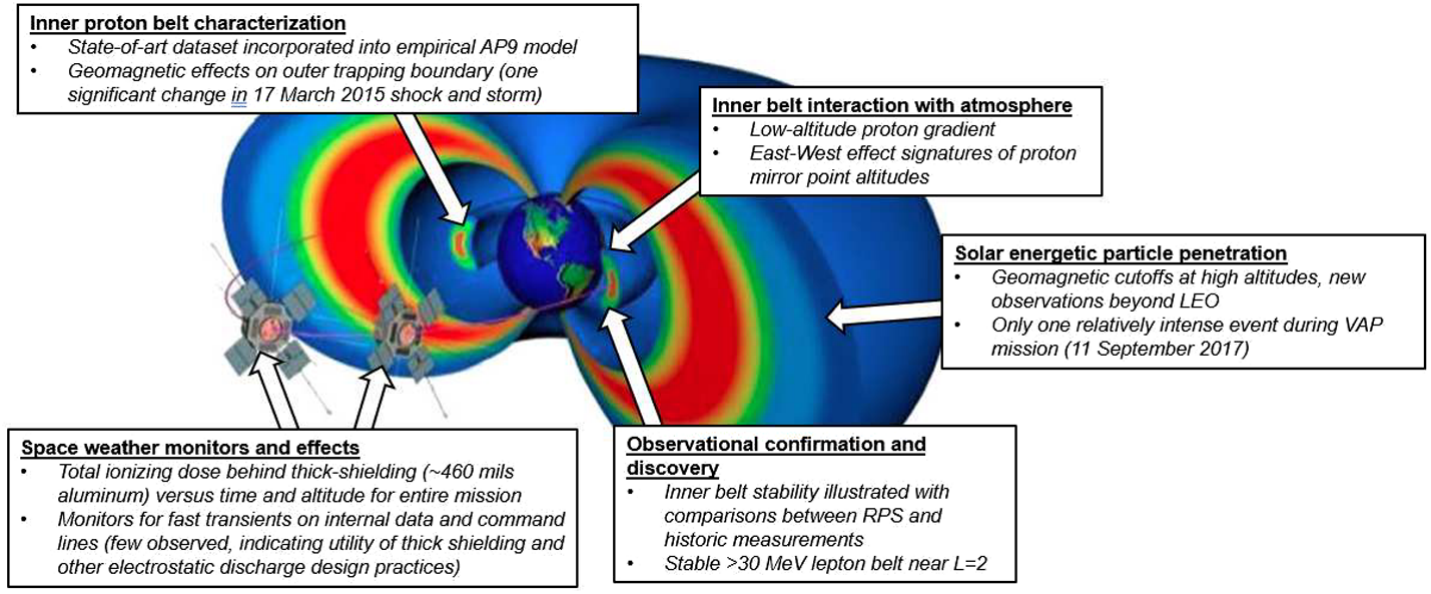
Graphical overview of RPS science and applications objectives and findings

Figure 3 qualitatively illustrates the phenomena that we targeted with RPS as well as other findings in relation to the mission orbit. Mission lifetime turned out to be the driver for whether we could close on some science questions related to transient radiation belts and solar particle events. In Sect. 2 we summarize what we learned from the investigation, emphasizing that in these (and likely other topics that we did not consider) there are probably significant treasures in the RPS data yet to be found. Our starting point is a brief review of the sensor performance and data products.

### Instrument Performance Summary

RPS met our level-1 project objectives. The pitch angle and spatial coverage provided by Van Allen Probes enabled us to collect the inner belt data that we needed for the AP9 empirical model.

The only anomaly that impacted the RPS Level 1 and 2 instrument performance requirements (Mazur et al. 2012) occurred within the microchannel plate/photomultiplier (MCP/PMT) on the RPS-A sensor. The MCP/PMT was a planar device that converted ultraviolet Cherenkov radiation into electrical signals for measurements of the amplitude of Cherenkov light. Beginning on 7 January 2016 the RPS-A MCP/PMT lost its ability to maintain the nominal −1650 volts bias across the microchannel plates. The device had shown signs of instability for short intervals prior to the failure (23 December 2014 and on 12 September 2015) but had recovered from those episodes without any intervention.

Our forward-backward proton discrimination relies on Cherenkov amplitude. We used the several years of concurrent RPS-A and -B operations to derive an empirical correction for the RPS-A fluxes (O’Brien et al. 2021a). The results were less accurate than the processing that used Cherenkov light. The final (v3) data products include the correction from 18 January 2016 through the end of the Van Allen Probes-A mission in October 2019.

After January 2016 we recommend using RPS-B alone for statistical analyses that involve fluxes accumulated over a fraction or more of a single orbit. The corrected RPS-A fluxes are of course available and can be applied to studies that require higher time-sampling provided by the two Van Allen Probes.

### Calibration

RPS calibration consisted of ground-based muon and proton testing of detector subassemblies; ground-based laboratory tests of the electronics and proton accelerator tests of the engineering model and both flight models; concurrent Geant4 modeling that included as-built performance of the silicon detector and Cherenkov subsystems; and in-flight comparisons of the two flight models with each other.

Throughout our investigation the Geant4 model was a living digital twin of the RPS physics hardware (see Jones et al. 2020 for a review of the digital twin concept). The model played a critical role in understanding the instrument’s performance for engineering applications, anticipated science, and discoveries.

Once on-orbit we also compared RPS to the REPT sensors as well as to historical inner belt measurements. The comparisons to other sensors were for reference and did not result in any adjustments to the basic Geant4 model response that we used for assigning proton incident energy and in deriving the higher-level energy channel assignments. We did use cross-correlations of RPS-A and -B to correct the RPS-A data to account for the failure of its Cherenkov system in January 2016 (O’Brien et al. 2021a, 2021b).

Section A.2 contains the details of the adjustments to the model. Most of the post-launch adjustments occurred within the Cherenkov subsystem. This subsystem had many free parameters related to scintillation, light absorption, and other aspects of light transmission that could not be otherwise determined from first principles. We also used the model to account for the $\sim25\%$ gain reduction in the RPS-B Cherenkov system. RPS included a command to raise the MCP/PMT high voltage; prior to flight we wanted that ability in case of unforeseen performance issues. We chose to change the model rather than command the high voltage because of the relatively gradual gain loss, coupled with the primary use as a direction discriminator and the ease of fine-tuning the model to fit the observed gain.

In summary, the RPS data products resulted from a comprehensive calibration campaign that started with engineering models and ended with insights from the flight data. We focused on the primary objective of the inner belt proton environment. In one of the science highlights below we pushed the model to provide information about RPS response to ultra-relativistic electrons. We do not have accelerator data for benchmarking the electron simulation. We argue in Sect. 2.2.3 that the weight of the observations regarding high-energy trapped leptons is sufficient to warrant new investigations with sensors that are designed for that particle species.

### Data Products and Levels

The primary data from RPS reflect our primary mission: proton flux in the inner Van Allen belt. Our processing focus was on those protons and actions we needed to take to ensure their collection and processing to higher level with all necessary instrument livetime and other corrections.

Prior to the mission start we had anticipated secondary science on solar energetic particle events. There are several solar particle events in the mission dataset, and one of these events had significant enough flux for science analysis as described in Sect. 2.

Future analyses can further exploit secondary science and engineering information from RPS: for example, detector singles rates; dosimeter total dose measurements, and the record of fast transients on the command and timing interfaces between RPS and the RBSP vehicles and factoring those engineering aspects with coincident measurements of the proton and electron environments. The detector singles rates could extend the RPS measurements to lower proton energies (or be calibrated for ionizing dose to provide another dose-depth curve from the mission) but would require additional modeling to understand their response.

We analyzed and produced RPS data in four separate levels, level 0 through level 4. Table 1 provides a summary of the RPS data products by level. Level 0 data originated in the RBSP MOC as files containing one full mission day of payload telemetry packets (PTPs). We converted the PTP files to NASA CDF format for wider use. There are separate PTP files for RPS rate and housekeeping data and for direct event data.

**Table 1:** List of the RPS data levels and brief descriptions of their contents

Data level	Name	Contents
0 (L0)	Level 0 Data	RPS PTP/CCSDS packets (decoded in CDF version, includes raw space weather data)
1 (L1)	Level 1 Data	Nearly all L0 data, UTC, energy/photon deposits, singles and coincidence rates, s/c location, RPS boresight vector, magnetic field vector, estimated incident energy/angle, dead times (including quota effects), space weather products
2 (L2)	Energy Spectra	UTC, flux versus energy spectrum (once per ∼5 degrees rotation), pitch-angle and full magnetic coordinates and direct events
3 (L3)	Energy-Angle Spectra	UTC, energy-pitch angle spectrum (once per minute), magnetic coordinates
4 (L4)	Global Maps	UTC, flux vs E/*α*_eq_/L_m_, flux vs E/K/Φ, flux vs E/K/h_min_ maps (once per day, once per orbit leg)

Refer to Sect. A.1 for additional details including file name convention and an overview of file contents.

### Summary of What You Need to Know to Use the RPS Data

For most science questions, the higher-level RPS data, like other charged particle measurements from Van Allen Probes and earlier missions, can readily be applied in science analyses. Starting in January 2016, we recommend using only RPS-B for detailed studies involving the proton flux data products that do not require coverage by both probes.

Further analyses of phenomena that we have examined during the mission and highlighted below (low-altitude gradients; solar particle access; time-dependent features of inner belt proton trapping; transient geomagnetic storm effects) are likely readily achievable with the processed and archived RPS dataset. Because RPS was not a typical range-energy charged particle telescope with out-of-geometry vetoes, phenomena like the galactic cosmic ray proton spectrum (Sect. A.4) and science from the RPS detector rates would require alternative data processing.

## Science and Applications Review

The Van Allen Probes orbit not only provided the coverage of the inner belt that we required, but it also enabled us to use RPS to characterize other aspects of trapped high-energy protons and other particles with signatures of the influence of the Earth’s atmosphere and geomagnetic field. From the gradient of the low-altitude inner belt protons to access of solar energetic protons, the RPS dataset reflects a wide variety of heliospheric phenomena that has a high potential for further research and exploitation.

### RPS Primary Objective

We designed and hosted RPS on Van Allen Probes to provide data for the AP9 climatology model of the proton radiation belt. A climatology model represents the mean, worst case, and other statistical properties of the radiation belt as a function of location, energy, and angle of incidence. The predecessor model, AP8 (Sawyer and Vette 1976), contained few proton observations above 50 MeV, and none above 150 MeV even though 43 separate space missions contributed data to the model by the time of its last update in 1976. AP8 was an extrapolation for satellite components that are shielded by about 3 inches or more of material. Spacecraft that need to operate in the inner belt, especially at altitudes above typical low-Earth orbit, would need a careful accounting of the radiation environment to make design decisions for radiation effects. RPS was built to replace extrapolation with observation in support of those design decisions.

Prior observations (e.g. Gussenhoven et al. 1993, 1996) as well as theoretical models of the cosmic ray albedo neutron decay (CRAND) process, suggested that the inner belt proton environment with above $\sim80~\text{MeV}$ is relatively stable. Stability enabled us to take a statistical approach to the RPS climatological observation mission: the instrument would not need to suppress Poisson counting statistics in every time sample, but could accumulate for days, weeks, or even months in adiabatic invariant coordinates.

We knew that there can be shorter-term variations in the inner belt from interplanetary shocks at its outer edge and atmospheric losses at lower altitudes. There was a trade between collecting power and associated design cost and interest in capturing shorter-timescale phenomena, not all of which were guaranteed to occur during the Van Allen Probes mission. Although RPS has much in common with the other particle sensors on Van Allen Probes, we chose the climatological mission as its primary objective. As a result, RPS had no burst mode or any other operating mode that depended on specific mission campaigns or events of interest; we powered on at the start of the mission and did not change operations. Nonetheless, as we discuss below, we were able to capture interesting changes in the inner belt, one being losses at the outer edge associated with the 17 March 2015 interplanetary shock.

At approximately the same time as the launch of Van Allen Probes, the AE9/AP9 team released the first version of the model (Ginet et al. 2013). Version 1.0 included many data set updates since the release of AE8/AP8 as well as new model capabilities such as space weather dynamics and model uncertainties. RPS data were not incorporated into AP9 until v1.5 (O’Brien et al. 2018a, 2018b) when enough on-orbit data were available to compute climatological quantities.

When the modeling team released v1.0, it was unclear how to extrapolate from the existing observations in the 50–250 MeV energy range up to higher energies. The RPS data resolved that ambiguity, providing satellite designers greater confidence in AP9 flux specifications and extending the energy spectrum up to $\sim1$ GeV.

**Fig. 4:**
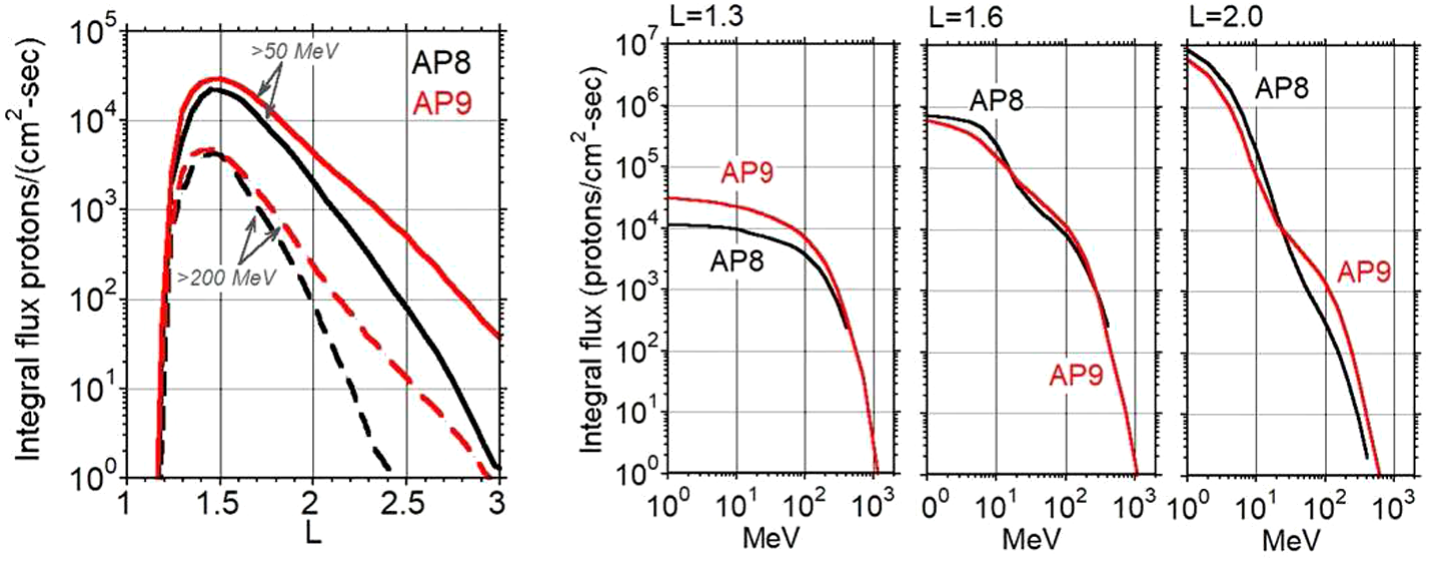
Comparisons between AP8 (minimum) and version 1.5 of AP9. Both models are mean environments at a specific point in space to yield near-equatorial fluxes (0 degrees latitude, 17 degrees longitude)

A common question is “How different is the new AP9 model from AP8?”. Figure 4 shows that in some places and at some energies the fluxes differ only by tens of percent or less and in others they differ by larger factors. The radial profiles in Fig. 4 show that above 50 MeV all the new measurements in AP9 have higher intensities above $\text{L}\sim 2$. Remember that these are comparisons between mean environments, with nothing besides the mean environment available from AP8. There is no single answer to the question of which model is more or less intense. AP9 is an improved characterization of the actual environment because of its statistical nature, its larger empirical database, and the benefits of the RPS energy and pitch angle coverage.

An implicit corollary to questions about which model is better is the observation that many satellites have been successfully designed to AP8. This has usually been done with margins applied to account for unknown uncertainties in the environment and in the system. Arbitrary margins aren’t needed with AP9, allowing for the application of margin where it should be applied: at the system and electronics parts levels, not on the external environment (Ladbury and Carstens 2021).

The model development team will release another version of AP9 that includes the RPS data set from the entire mission (v1.5 included data from 2012 to 2016) with final calibration and cleaning. The modeling effort will expand its capabilities by including international space missions and partners that have coverage of the inner belt and is taking on a new name: IRENE (International Radiation Environment Near Earth). The IRENE team expects to continue to develop the climatology models, adding solar energetic particles and new data sets from current and future flight missions.

### Selected Mission Highlights

The Van Allen Probes orbit provided the opportunity to sample a wide range of near-Earth phenomena. Through the lens of a very specific sensor like RPS, these phenomena often look different compared to lower-energy particle spectrometers, or are not present at all except in extreme events. In some cases, like solar particle events, the particle environment has to exceed typical intensity levels in order to yield statistically significant measurements.

If one thinks of every Van Allen probes traversal of the inner belt as a storm, then the total RPS dataset contains about fifteen thousand storm events. As we show below, our target storm environment is relatively constant in time so perhaps the storm analogy is not the best. One thing we did learn is that a sensor designed for a specific measurement often can surprise us when we look at other regions of the magnetosphere.

#### Comparisons to Historical Inner Belt Measurements

We had several historic inner belt proton results in mind when, prior to the Van Allen Probes mission start, we set as a goal to see if the CRAND process and the known secular magnetic field evolution could explain what we would be observing with RPS. As we compared some of the past measurements with RPS, we quickly realized two things: one, that some of the one-off historical glimpses of the inner belt intensity and energy spectrum were surprisingly accurate; and two, that indeed, the long-lifetime of the inner belt protons and our knowledge of the Earth’s magnetic field together tell a consistent story.

Many of the older datasets that overlap in energy and location coverage are not available in digital format. One is left with the record as published in the scientific literature. For the many sub-orbital flights that sampled the inner belt in the 1960s there is no spatial or magnetic field ephemeris other than what had been published. For the orbital satellites, two-line elements exist that allow one to compute the orbit using modern methods and magnetic field models.

Table 2 lists some of these historical glimpses of the inner belt that we considered as well as comments on the relevance of the older data.

**Table 2:**
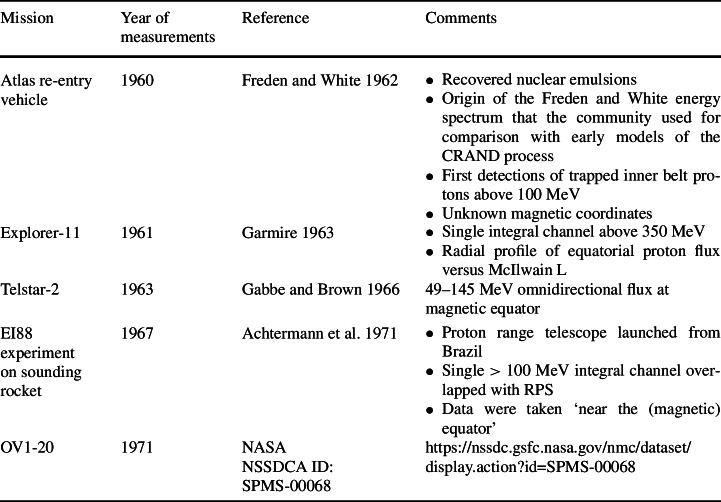
List of selected past missions with partial sampling of the inner belt that overlapped with RPS energy and spatial coverage

To illustrate the challenges and the successes of these comparisons, we highlight in detail here a dataset from the last mission in Table 2. The US Air Force Orbiting Vehicle 2- (OV1-20) launched on 8/7/1971 with eight payloads; one was a pair of battery-powered Cherenkov spectrometers designed and built by The Aerospace Corporation (W. Kolasinski was the Principal Investigator and N. Katz a senior engineer for the OV1-20 proton instrument; they also were part of the RPS engineering and physics team). The spectrometers used a fast coincidence between two thin scintillators and a thicker Cherenkov radiator (Lucite plastic or lead-fluoride) to yield two integral channels that overlap with RPS ($>65$ and $>550$ MeV) as well as three differential energy channels. We compared RPS with the spectrometer that used a Lucite radiator because of suspected gain changes in the other sensor.

The internal high voltages would have normally required a long outgassing period before operating the sensor. However, the host system had a low initial perigee (136 km) that led to a short 21-day mission life. As a result, the sensor used a pressure vessel and battery power to realize its 8-day mission with data acquisition only occurring in high-radiation portions of the orbit (that is, the inner belt). A fast 12-rpm spin rate and good statistics allowed the sensor to adequately sample a wide range of proton pitch angles.

The sensor developers had always intended to re-do the OV1-20 spectrometer for a longer-lived mission in the future. They archived the data but did no specific analyses of them with the thought that only a longer mission warranted the attention. That hoped-for longer mission with a version of the Cherenkov instrument failed on launch. Science priorities changed over time and it wasn’t until 40 year later that Van Allen Probes included a high-energy proton sensor designed to work in the challenging inner belt.

**Fig. 5:**
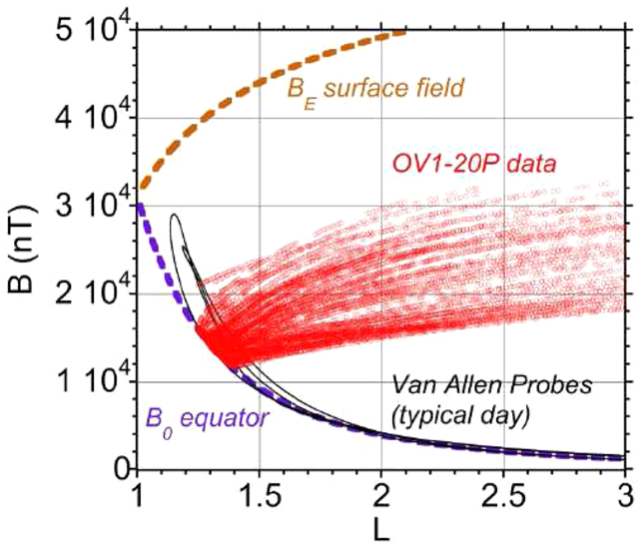
Mapping of the OV1-20 and Van Allen Probes orbits into B-L space. Note the overlap between the two missions at the magnetic equator well into the heart of the inner proton belt. This was the prime motivation to reanalyze the OV1-20 proton spectrometer data in the context of Van Allen Probes

A. Vampola digitized the original database, used sensor design information to perform corrections, and published the results to the NSSDC (Data ID SPMS-00068, Vampola n.a.). As Fig. 5 shows, there was good overlap between the OV1-20 and Van Allen Probes in coverage of the inner belt magnetic equator between L of 1.2 and 1.4; this is where the peak intensities lie for the highest inner belt proton energies.

The next step in this comparison was to fly OV1-20 through an inner belt environment as specified by the RPS data. The process was: use several months of RPS data to accumulate unidirectional flux versus equatorial pitch angle in 0.01 wide L shell bins and 1-degree pitch angle bins; use the mirror equation (Roederer 1970) to map the perpendicular flux everywhere down the field line; propagate the OV1-20 orbit to calculate the magnetic field coordinates (L shell and local field intensity) for every datapoint in the OV1-20 dataset; output the RPS-derived perpendicular flux at those OV1-20 locations. The process resulted in the time-history from the older mission (epoch 1971 in the case of OV1-20) being shown along with what RPS would have specified as a proton belt at that time.

**Fig. 6:**
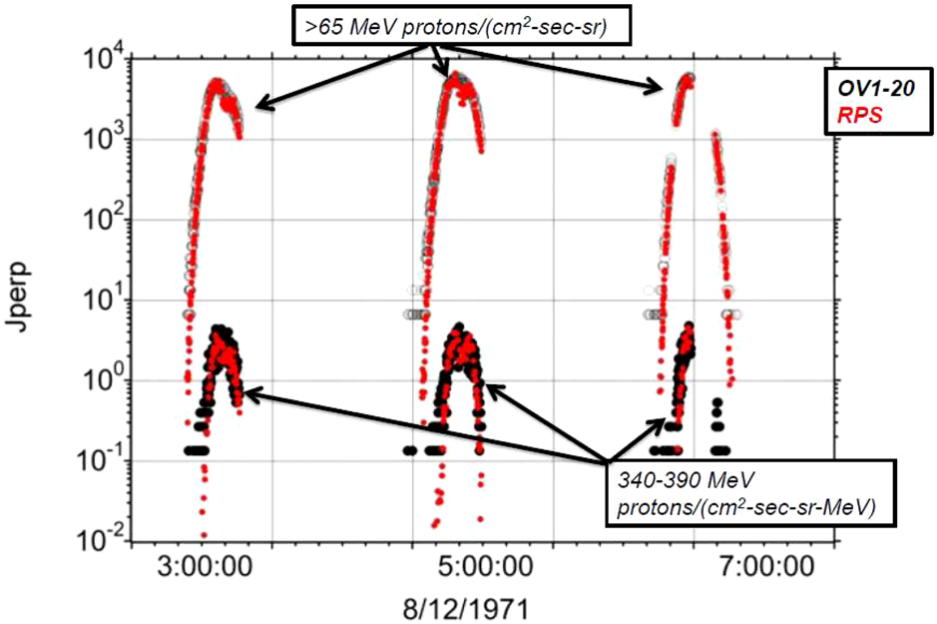
Mapping of the OV1-20 orbit into an empirical inner belt as determined with RPS measurements from 40 years later

Figure 6 is an example of one integral and one differential OV1-20 proton time-intensity profile for three representative passes through the inner belt in 1971. At the same times we show the empirical RPS inner belt equatorial fluxes where we re-sampled the RPS data to match the OV1-20 energy channels. The figure shows that the ephemeris we calculated adequately organizes the OV1-20 data and that the RPS-derived inner belt intensities agree with OV1-20, sometimes within 20%.

**Fig. 7:**
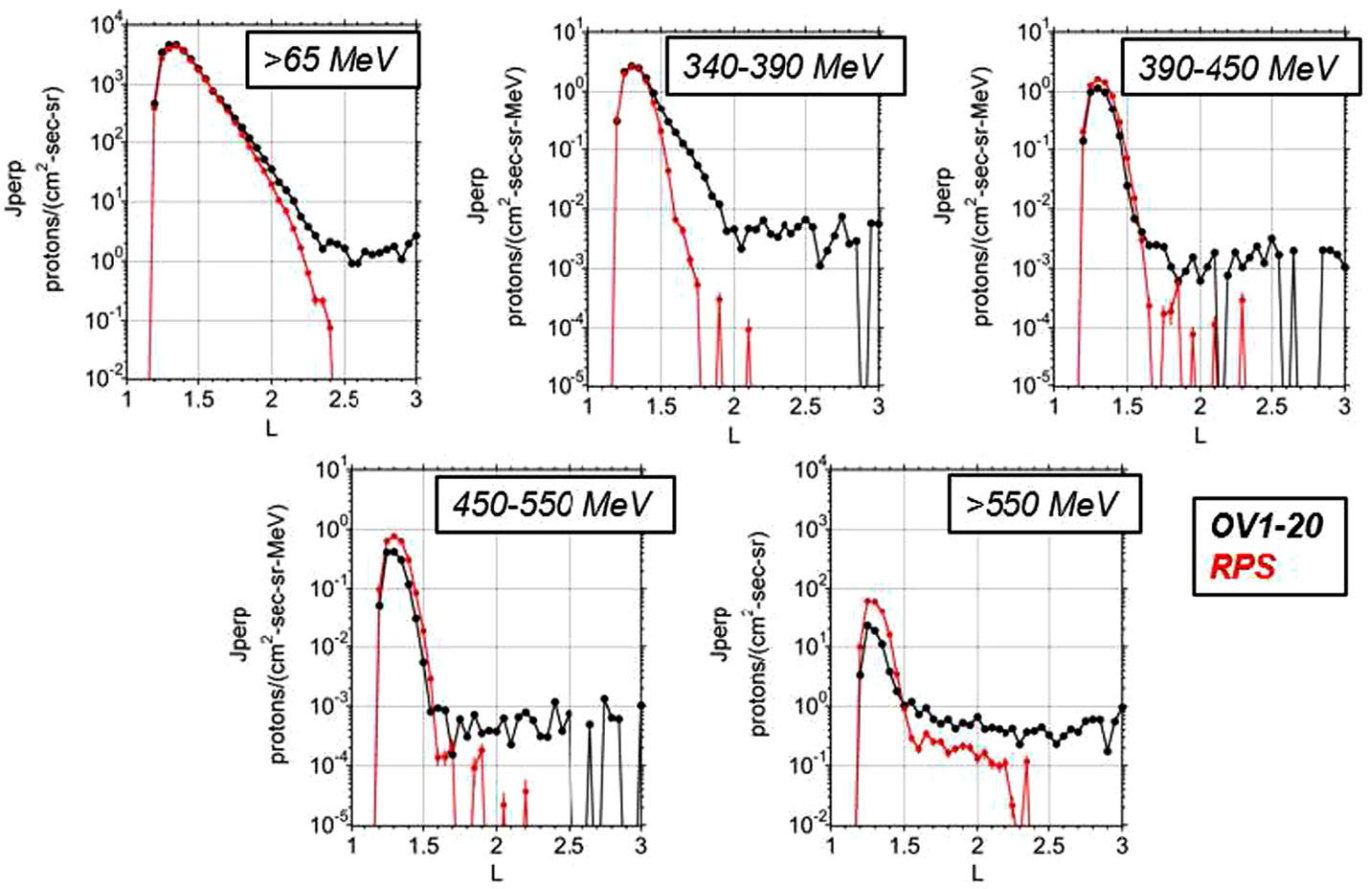
Montage of all OV1-20 equatorial proton flux data compared with RPS

Figure 7 synthesizes all the data from the entire eight days of OV1-20 compared to RPS at the same energies and McIlwain L shells. At peak intensities the two datasets agree except for the two highest OV1-20 energies. At higher L, some of the channels match and some do not. All the OV1-20 channels have flat extensions of flux out to $\text{L}=3$ while RPS does not, possibly indicating some background from residual electrons in OV1-20. The mission flew near solar maximum and we would have expected the GCR proton flux at these energies to have been about a factor of 10 below these flat extensions. We also note that Vampola wrote that the OV1-20 data were not corrected for any accidental coincidences (Data ID SPMS-00068).

As we will demonstrate in the next section, we expect time-dependence in the outer edge of the inner belt, so perhaps in that context the core inner belt comparisons should carry the most weight. By that measure we see an incredible demonstration of the stability of the inner belt at these energies. A later section discusses the apparent tail above $\text{L}\sim 2$ in the $>550$ MeV RPS channel.

**Fig. 8:**
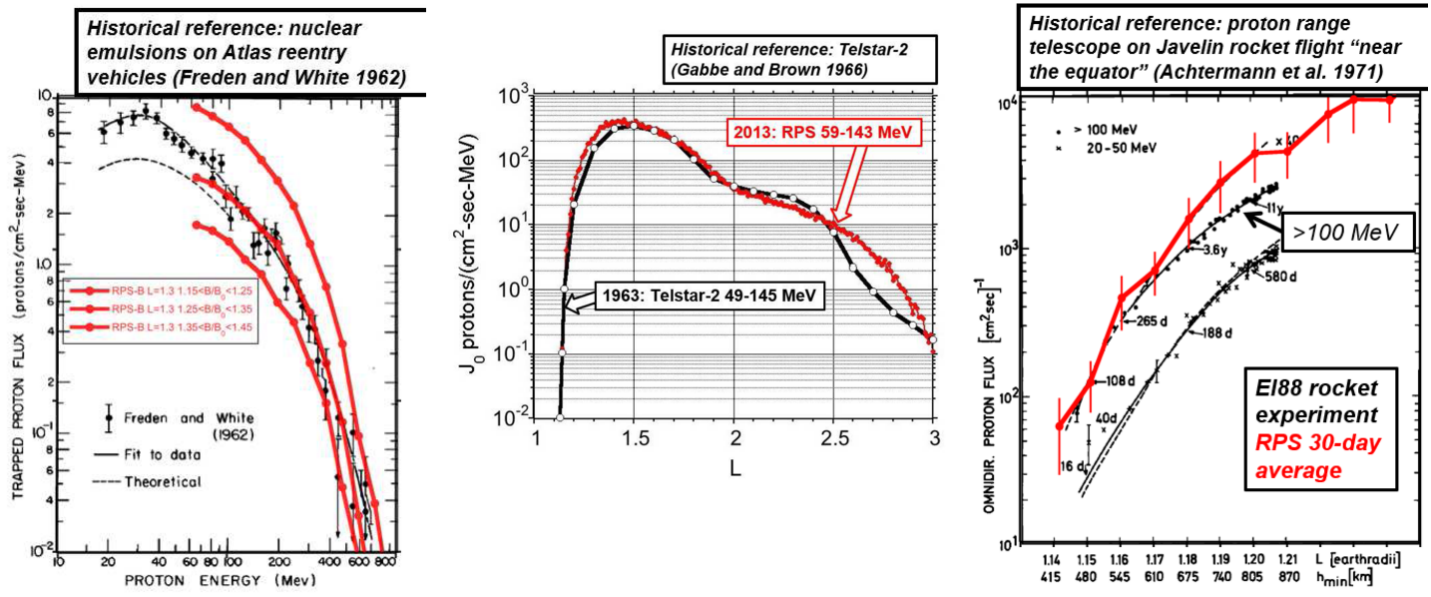
RPS proton fluxes (red) overlaid on a selected set of published inner belt fluxes (black)

The other historic inner belt measurements in Table 1 were available as graphics in scientific publications. Figure 8 compares RPS measurements with the published data where, for the non-orbital missions, we showed the equatorial proton flux to compare with the Achtermann et al. (1971) radial profile and a range of off-equatorial fluxes to compare with the Freden and White (1962) energy spectrum. The comparisons were not sensitive to the choice RPS accumulation intervals except for the case of the Telstar-2 radial profile (Gabbe and Brown 1966) discussed below.

In every case, we find reasonable agreement between the historic and Van Allen Probes era inner belt intensities. It is also possible that a change to the inner belt topography account for the difference between the Achtermann et al. (1971) data and the RPS measurements, although the former were from a single suborbital snapshot of the inner belt. Now consider Telstar-2 and RPS, two samples of the inner belt taken from orbiting platforms over a fifty-year interval. The Telstar-2 radial profile was within a factor of two of the RPS profile up to $\text{L}\sim 2.5$, beyond which there was a larger divergence. Given the good correspondence between the data at lower-L where obtaining a high-quality measurement is most difficult because of the penetrating proton background, this suggests the difference at higher L is real and is a result of a time-dependence to this less-stably trapped portion of the inner belt. The next section illustrates this was probably the case because a similar change occurred during the Van Allen Probes mission.

#### Changes to Inner Belt Topology

In our routine data processing checks, historical comparisons, and AP9 model development, we can say with confidence that there were no changes to the peak ($\text{L}\sim 1.5$) inner proton environment above 60 MeV as large as the changes above $\text{L}\sim 2.5$ discussed here. Even so, we believe that relatively simple analyses of the entire mission dataset might reveal subtle changes, especially in regions where the atmospheric losses or weaker magnetic field help govern the instantaneous proton population.

**Fig. 9:**
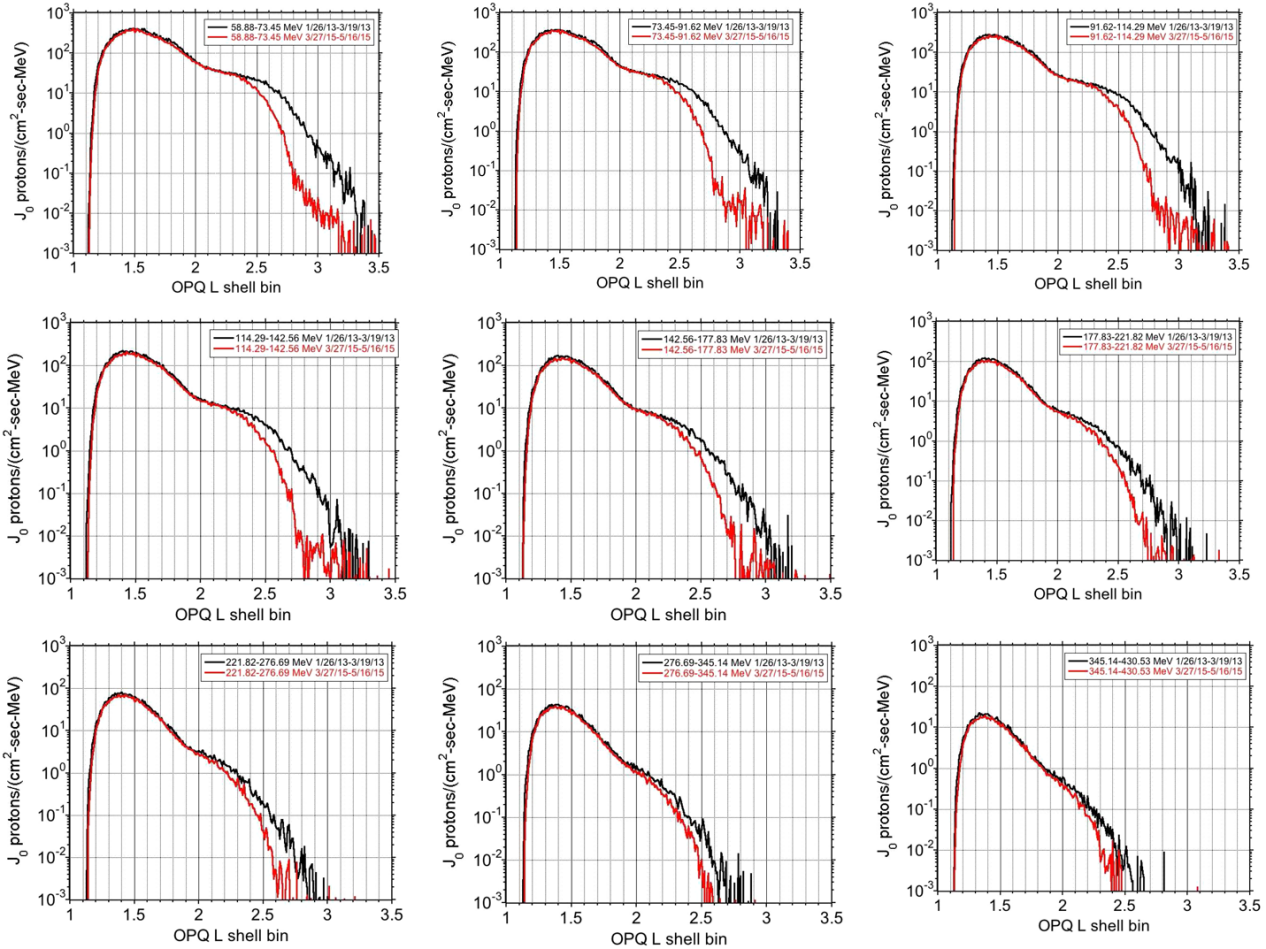
Equatorial proton fluxes prior to (black) and after (red) the 2015 March 17 interplanetary shock binned in the Olson-Pfitzer Quiet magnetic field model (see Sect. A.1)

A dramatic change to the outer trapping region occurred with the interplanetary shock of 17 March 2015. This event led to a series of observational and modeling studies on the prompt acceleration of ∼several MeV electrons (e.g. Kanekal et al. 2016; Hudson et al. 2017). Figure 9 summarizes the changes that we observed using approximate 2-month averages of RPS data well before the shock and soon afterwards.

The trapped proton intensity decreased by a factor of $\sim30$ at the lowest energies at $\text{L}\sim 3$. Selesnick et al. (2016) showed similar decreases below $\text{L}=2.8$ and below $\sim70$ MeV. The results in Fig. 9 have not yet been fully exploited in the sense of modeling the losses with empirical shock and modeled storm parameters, and the expected recovery of flux through radial diffusion. These observations of the magnitude of the decrease versus L suggest several areas for future research to test our understanding of the losses and replenishment of the outer edge of the inner belt.

#### Discovery of a Lepton Belt

We knew that the outer edge of the inner belt was the interface with a region that has housed new radiation belts with origins in interplanetary shock events (e.g. Blake et al. 1992) as well as reservoirs of other samples of matter from the solar system like anomalous cosmic rays (e.g Cummings et al. 1993). New electron radiation belts also appeared in this area during the Van Allen Probes mission (Kanekal et al. 2016) as well as during the last solar cycle (Looper et al. 2005).

**Fig. 10:**
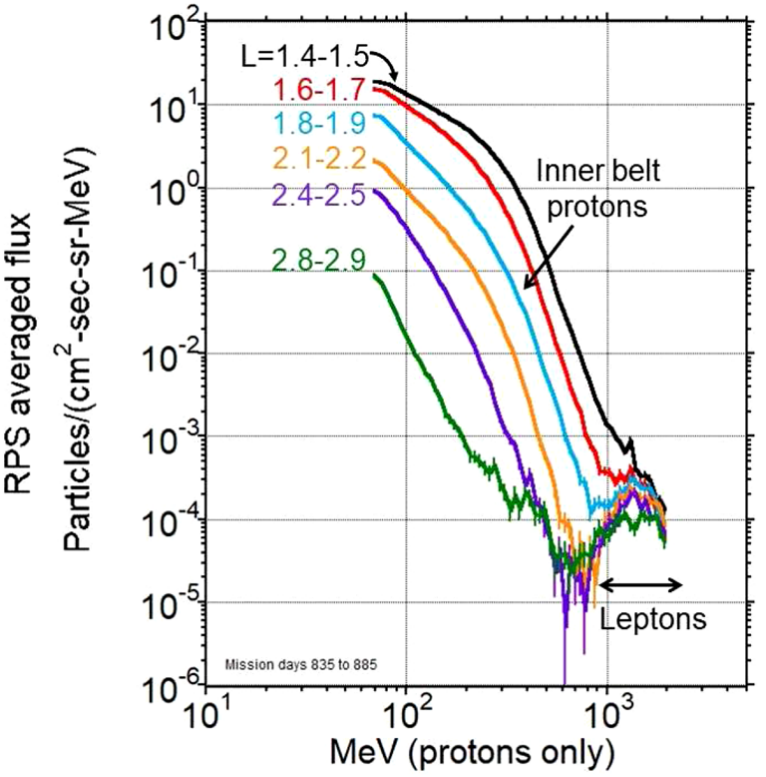
Spin-averaged local particle flux averaged over a fifty-day interval beginning in mid-December 2014. The turn-up in the energy spectra beginning at proton energy $\sim800$ MeV is the manifestation of $>30$ MeV leptons as described in this section

It was natural for us to survey the outer edge of the trapping region using RPS as intended for protons. Our standard data processing exploited the directionality afforded by the Cherenkov light subsystem and the thick inert shielding behind that subsystem to minimize the signals from backwards-going protons that penetrate the telescope. We assembled high resolution energy spectra like the sample in Fig. 10 using the standard data selection methods. We were surprised to see a clear turn-up of the energy spectrum beginning near $\text{L}\sim 1.6$ that exceeded the galactic cosmic ray flux up to L shells as high as $\text{L}\sim 3.0$.

The population of interest here is labeled leptons in the figure. We should explain why we refer to them as leptons and not electrons. RPS cannot differentiate electrons from positrons. In this section we present simulation results with incident electrons when positrons would have identical responses. Neither can any of the observables we show differentiate between these lepton types. We chose to call this population leptons because several candidate sources could produce both electrons and positrons.

Getting back to the observations, the first puzzling aspect of these signals was the assignment of high ($>800$ MeV) energies based on the combination of Cherenkov light and solid-state detector amplitudes. Ignoring the question of what mechanism would yield a high-energy proton population so far from the CRAND source, was it possible to stably trap protons at these energies between $\text{L}\sim 2$ and 3? Model calculations that account for the gyroradius and field-line curvature (e.g. Selesnick et al. 2013) don’t explicitly rule out trapping of an 800 MeV proton that resides at the magnetic equator at $\text{L}=2.5$. In our case, the bulk of the signal in Fig. 10 resides above 1 GeV and that energy is well above what Selesnick et al. (2013) modeled for stable trapping for any equatorial pitch angle proton. We next turned to other diagnostics that the sensors provided to identify these particles.

**Fig. 11:**
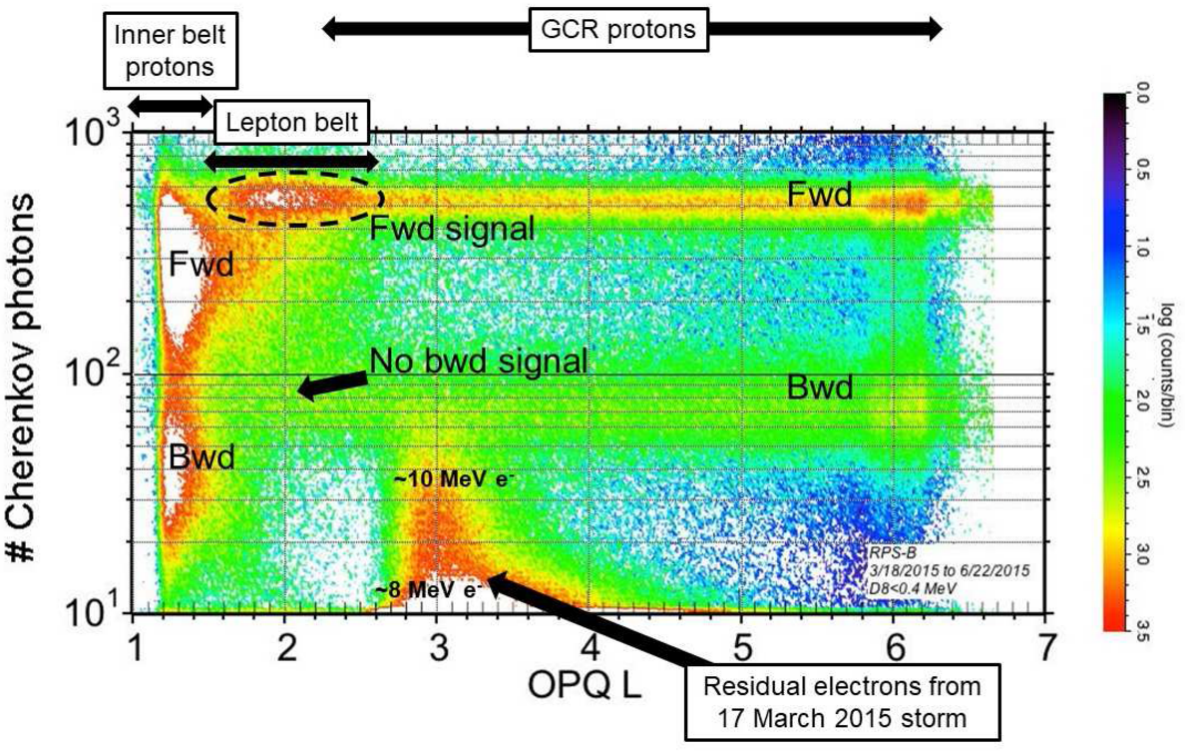
Several-month accumulation of coincidence events that qualify as minimum-ionizing in RPS. Several distinct populations appear in this format of the RPS data as well as the overall forward- and backward-going morphologies of the inner belt and galactic cosmic ray protons (labeled in the figure as Fwd and Bwd, respectively)

The Cherenkov light amplitude turned out to be a critical signature for identifying this population. Looking at the amplitudes of coincidence events in the RPS dataset is not the most straightforward way of identifying various particle populations, but it does illustrate one key argument against the anomalous particles being protons. Figure 11 is a several-month accumulation of pulse-height events with small solid-state detector amplitudes that correspond to minimum-ionizing particles. We plot their Cherenkov light amplitude versus L shell as a two-dimensional histogram of events per L and amplitude bins.

The sensor design biases against light from backwards-going protons. A proton requires about 200 MeV to traverse from the back of RPS and trigger the front A1 detector. The design does not entirely eliminate light from backwards protons, but lowers its amplitude by about a factor of 6 compared to forward-going protons. Figure 11 shows this reduction in amplitude and overall number of events cleanly in the inner belt region. Backwards-going GCR proton flux has similarly lower light amplitude. Electrons from the 17 March 2015 geomagnetic storm appear in the figure with low light amplitude. Our model calculations indicate that we are seeing electrons between about 7 and 10 MeV in this display at $\text{L}\sim 3$. Our level 2 and greater proton data products do not include these electrons. Features of the GCR counts in this display above $\text{L}\sim 5$ result from periodic changes in the sampling of higher L shells in the Van Allen Probes orbit. Sampling of L shells in the we are discussing in this section was uniform in comparison.

Now turn to the feature labeled as the lepton belt in Fig. 11. Two important observables are: there is no corresponding backward-going signal in the same L range at least in a forward/backward intensity ratio range as seen in the inner belt and GCR proton populations; and the Cherenkov light amplitude extends to slightly higher amplitude than provided by the minimum-ionizing protons in the GCR. For reference, the forward-going lepton signal highlighted in Fig. 11 is about a factor of three higher than the cutoff GCR intensity at $\text{L}=2$ as will be shown in detail below.

We needed to identify a particle that had no apparent backwards-going signal, was still minimum-ionizing in the silicon detectors, and was able to produce higher Cherenkov light amplitude than forward-going protons. We turned to our Geant4 model and investigated the response of the RPS physics package to higher-energy electrons.

**Fig. 12:**
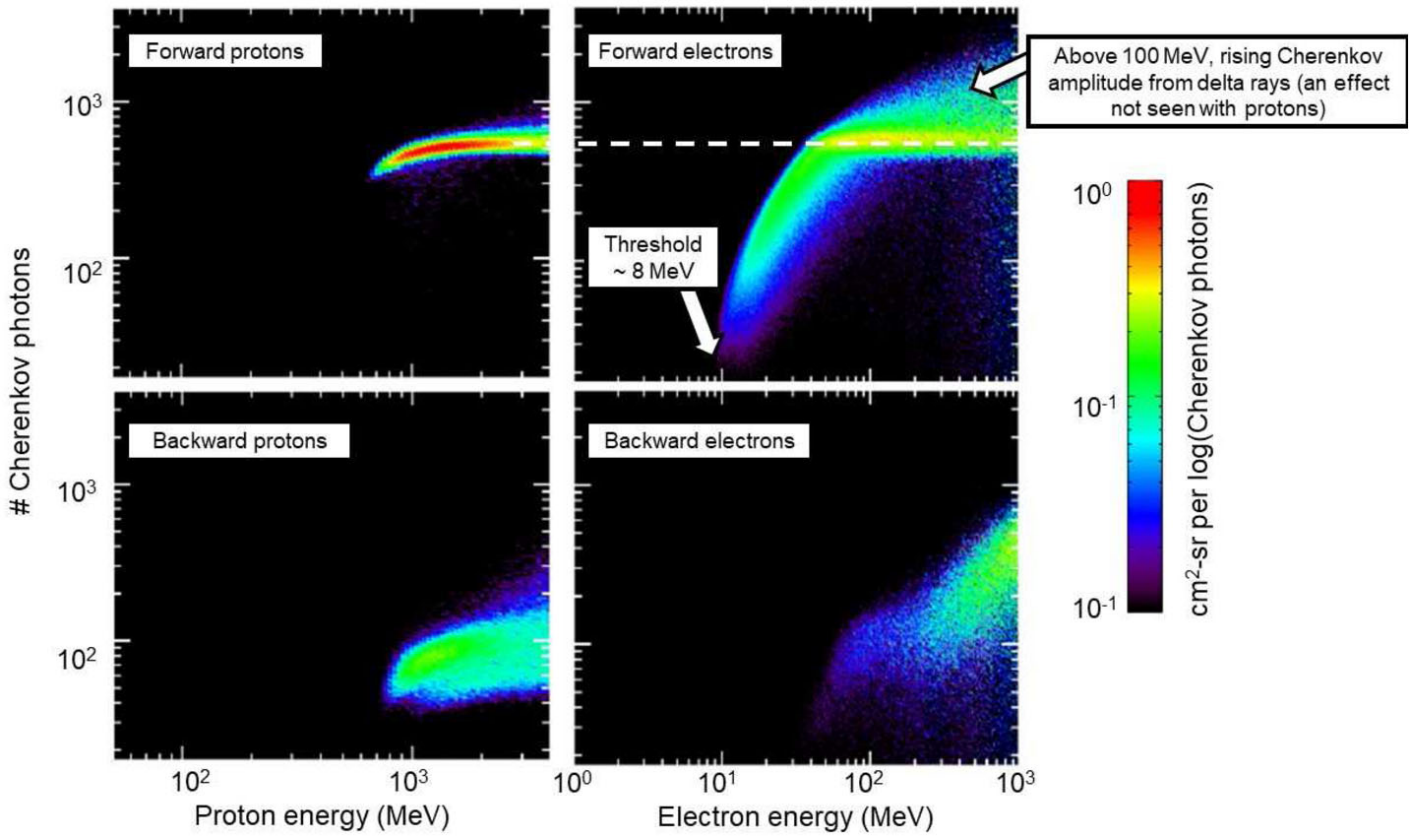
Simulation of forward and backward-going protons and electrons in RPS. Events selected to have minimum-ionizing signatures in the solid-state detectors (in this case between 0.27 and 0.33 MeV deposit; see Looper et al. 2021a)

Figure 12 shows the high-energy electron simulation of Cherenkov light compared to protons after a selection for minimum-ionizing deposits in the solid-state detectors. We included the protons in the figure to show that electrons produce light with higher amplitude than protons; this extra light per incident particle arises from delta-ray production by the electrons in the RPS Mallory shielding and the magnesium fluoride crystal. Delta-rays add to the light output for both relativistic ions (e.g. Swordy et al. 1983) and electrons but the generation rate and additive effects are larger for electrons within the RPS energy ranges, consistent with our observations. It was more surprising that the simulation showed that for electrons to saturate the light output, they would require incident energies of $\sim30$ MeV and greater. Note also the threshold of about 8 MeV for forward-going electrons to appear as coincidence events.

In Fig. 11 we observed the remnants of an enhanced outer belt near $\text{L}\sim 3$ whose light amplitude didn’t exceed that for $\sim10$ MeV electrons. There is no comparable lower-light and therefore lower-energy signal in our $\text{L}\sim 2$ lepton belt population. Taken together with the presence of a saturated Cherenkov signal, these facts indicate that we are looking at a population that is at least 30 MeV with little to no flux at lower energies. RPS does not have the best electron resolution but we can say for certain that this population has a unique energy spectrum with little to no flux below 30 MeV.

We simulated a range of spectral forms and found that our light distribution is consistent with an $\text{E}^{-3}$ energy spectrum that begins at 30 MeV. Our observations are not a tight constraint on the spectral shape or how high in energy it extends beyond 30 MeV, but they do point the way to what a follow-on exploration of the population would be required to measure.

The gyroradius of a $\sim30$ MeV electron is about the same as a 200 MeV proton ($\sim520$ km) at $\text{L}=2$. The inner belt has an intense population of 200 MeV protons at $\text{L}=2$ that remained in place after the 17 March 2015 shock (Fig. 9), suggesting a stable trapping for high-energy leptons in this region.

Where did these particles come from? We have considered the options in Table 3. We did some in-depth modeling of the Jovian spectrum observed at 1 AU that yielded electrons in the right location, but we could not explain the lack of particles below $\sim30$ MeV (O’Brien et al. 2016). We list the other options for the record. There could be other potential sources besides these.

**Table 3:**
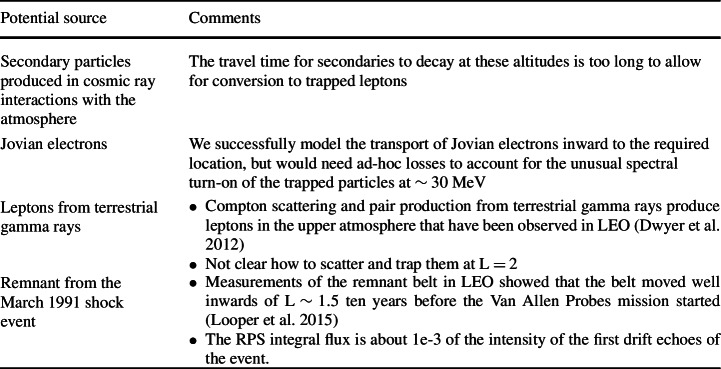
List of some possible lepton belt mechanisms and preliminary evaluation and comments on each one

**Fig. 13:**
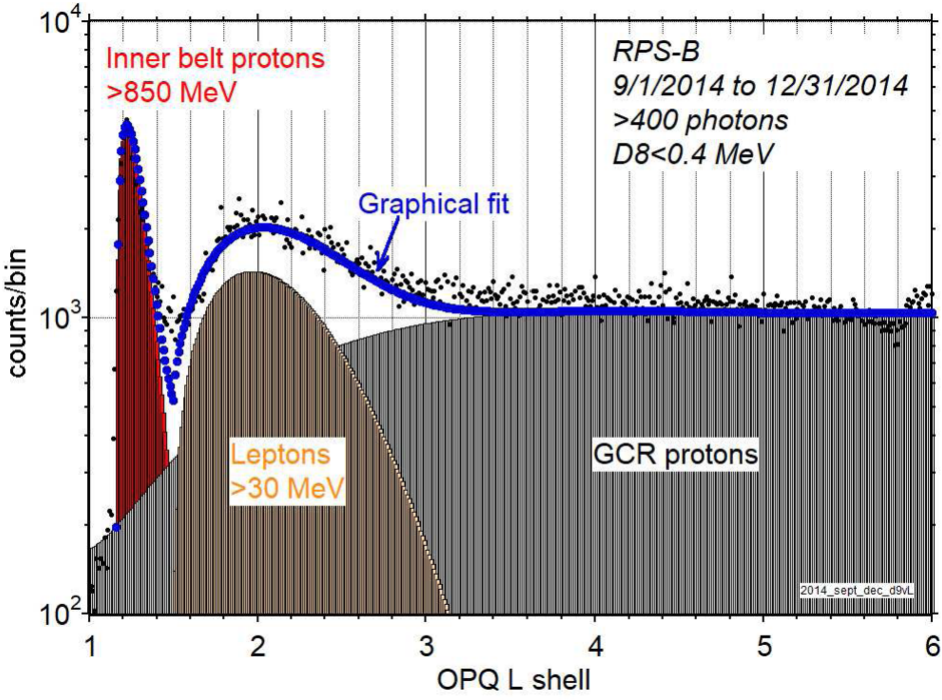
L-shell profiles of the inner belt, galactic cosmic rays (with the empirical geomagnetic cutoff from the SAMPEX mission), and the $\text{L}\sim 2\text{--}3$ lepton belt. The curve is a fit by eye to these three populations that add to form the observed radial profile

We have been able to rule a few things out based primarily on our inferences of the energy spectrum. There are more aspects of the population that could be brought to bear on other ideas and models of potential sources. These include the pitch angle distribution (not limited to equatorial particles, broader than typical inner belt protons; Looper et al. 2015) and the stability of the population across the entire Van Allen probes mission (no significant change in intensity during the first four years of the mission; O’Brien et al. 2016). Figure 13 puts the population into context with the inner belt and galactic cosmic ray protons as a way to set the stage for future investigations of this compelling population.

#### Solar Energetic Particle Access

Before launch we anticipated using RPS to help answer questions related to solar energetic particles (SEP). We looked forward to contributing to a Van Allen Probes dataset of proton measurements across a wide energy range similar to that compiled by Mewaldt et al. (2005). Studies of SEP sources, acceleration, and propagation have exploited elemental and isotopic composition (e.g. Leske et al. 2007), sometimes leaving the most abundant protons for other platforms to measure. We wanted to fill some of those gaps with RPS and the ECT suite. The SEP events would also have provided a convenient ‘standard candle’ for intercomparisons of our spectrometers with other proton sensors in the near-Earth environment in LEO, GEO, and at the moon.

The phasing of the mission with solar cycle and the relatively low-intensity SEP events observed at Earth in the past decade led to less solar particle research with RPS than we had anticipated. The NOAA list of solar proton events from GEO identifies more than 20 events above 10 MeV during the Van Allen Probes mission. RPS, being sized to focus on the inner belt with a relatively high energy threshold, detected few of these with enough statistics for any detailed studies. The 10–14 September 2017 event did have sufficient statistics in our energy range for analyses of the geomagnetic cutoff phenomenon.

O’Brien et al. (2018a, 2018b) approached the solar particle topic with a focus on geomagnetic cutoffs, both the models themselves as well as the utility of using cutoff measurements in LEO to say what the environment is at higher altitude. One new finding was that the spatial gradient at high-altitude, combined with the relatively large proton gyroradius, led to the L shell of the particle’s gyrocenter being a better organizer of the local intensity than the L shell itself. The same phenomenon occurs in LEO but there the spatial gradients are much smaller and the effect is often negligible.

Another finding was the inadequate ability of cutoff models to describe the cutoff variations that resulted from moderate geomagnetic activity during the 2017 event. Our single solar particle event suggested that that cutoffs measured in polar LEO are indeed useful indicators of the SEP environment in higher Earth orbit, but the reproducibility depends on the sensor field of view and energy response.

Transient solar particles revealed the importance of sensor response when it comes to protons with very large gyroradii. The inner proton belt provided more data on spatial gradients and the role of guiding centers that can be many tens to hundreds of kilometers away from the sensor.

#### East-West Effect and Low-Altitude Gradient

In 2013 we revisited the historic inner belt East-West effect with a study of pitch-angle resolved protons above 60 MeV (Mazur et al. 2014). This particular integral channel had good counting statistics for an initial study. At $\text{L}\sim 1.2$ the combination of large gyroradii and the scale height of the atmosphere leads to an asymmetry in the trapped proton flux depending on whether the particle guiding center is above or below the observer. At L shells above $\sim 2$ the anisotropy resulted from higher intensities at altitudes below RPS, not a surprise but not reported before. All these observations were consistent with the flux gradients that we observed and benefitted from the minimal backgrounds from out-of-geometry protons that RPS provided.

One new aspect of the general topic that we illustrated in 2013 was how the minimum altitude of the guiding center appeared to be reflected in the amplitude of the low-altitude anisotropy. This effect had been hidden in prior studies that time-averaged the East-West effect. One particular example that we showed revealed a beating of the East-West flux ratio that correlated with the minimum guiding center altitude; this meant that we were essentially able to sound the atmospheric scale height on the time scale of $\sim1$ minute over altitude ranges from 1000 to 1700 km. We know this signature is present throughout the mission data set. It has the potential to reveal changes to the atmospheric loss during geomagnetic storms and across a portion of the rising solar cycle.

The lowering of the Van Allen Probes perigees for mission disposal provided a chance to sample the inner belt flux gradient at altitudes much lower than the nominal 600 km perigee that we sampled during most of the mission. Altitudes much below $\sim400$ km are of scientific and engineering interest (e.g. Berthoud et al. 2022) yet have not been as thoroughly explored from LEO because of short orbital lifetimes. Some example missions that probed the inner belt below 400 km were the AMS-01 spectrometer on board a space shuttle flight in 1998 (Alcaraz et al. 2000) and the approximately first four years of the PAMELA investigation on the Resurs-DK1 mission (Bruno et al. 2021).

**Fig. 14:**
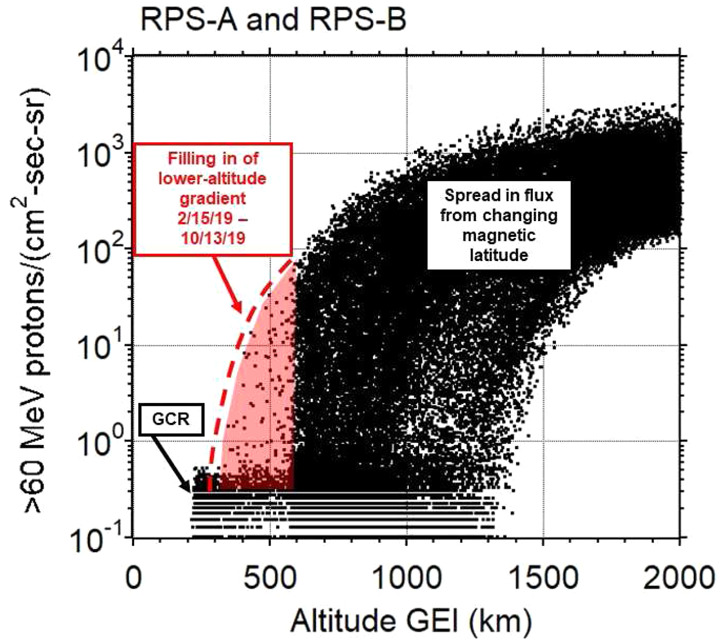
Five-minute averaged proton flux from the entire mission dataset to show the sampling of the inner belt altitude gradient below $\sim600$ km made possible by the perigee-lowering maneuvers in early 2019. The red dashed and shaded regions guide the eye to additional measurements after perigee-lowering maneuvers

Figure 14 shows a glimpse of the RPS measurements in the form of five-minute spin-averaged flux of $>60$ MeV protons for the entire mission datasets of RPS-A and -B where the bulk of the data are from the operational orbit. Beginning in February and March 2019 we started to sample lower altitudes as shown in the highlighted region. The spread in the data arises from varying magnetic latitude.

The target area of interest decreases in spatial extent as altitude decreases and that limits the amount of data we have, but the low background and pitch-angle resolution are unique. This is another aspect of the mission and dataset that holds potential for engineering and science research.

Turning to engineering, we next discuss auxiliary aspects of RPS that provided direct information about the radiation and spacecraft charging effects in the stressing Van Allen Probes orbit.

#### RPS Dosimetry

Each RPS sensor housed two microdosimeters (Mazur et al. 2011, 2012) within the RPS housing for not only monitoring the dose within the sensors in case there were issues that we thought might have arisen from total radiation dose (there were none), but also as a contribution to the space weather engineering output of the mission. After launch we performed detailed Geant4 modeling of the dosimeter response functions. Our analyses were more detailed than what we would expect of other systems or payloads that integrate one of these dosimeters. We had to take advantage of two aspects of the unique Van Allen Probes opportunity to illustrate the usefulness of the dosimeter design. One aspect was the relatively harsh radiation environment with passes near the magnetic equator in the inner belt. The other was access to accurate electron and proton energy spectra on the same vehicle for convolving with the dosimeter response. Typical accommodations of the dosimeter would not have the same external energy spectra to use for detailed study.

**Fig. 15:**
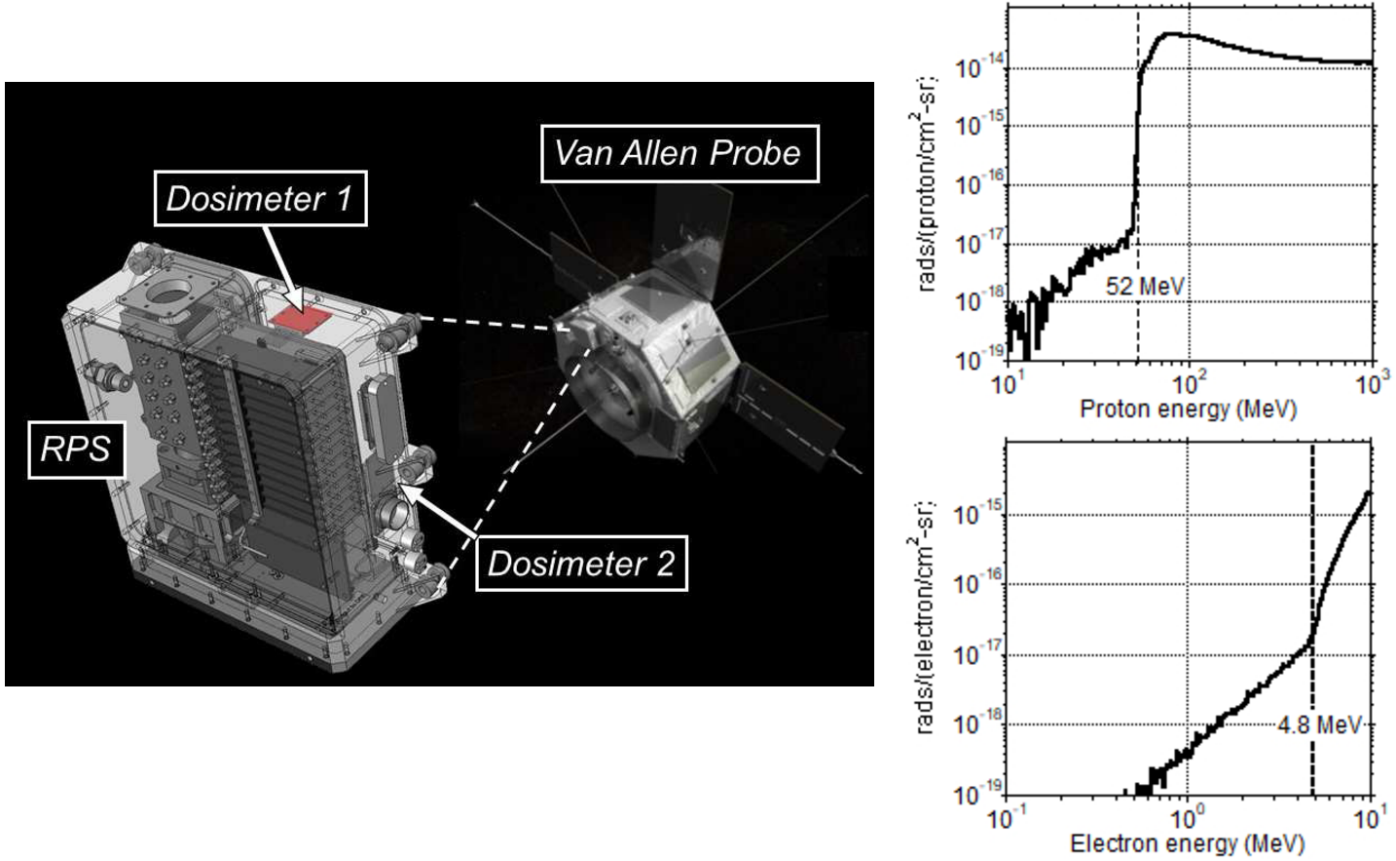
Transparent view of RPS showing the physical locations of the dosimeters labeled 1 and 2 in the RPS telemetry (left). We focus on the data analysis of Dosimeter-1 in RPS-B. Modeled response functions of Dosimeter-1 for omnidirectional electrons and protons (right). Van Allen Probe image courtesy of NASA

For unknown reasons only one of the four RPS dosimeters (Dosimeter-1 on RPS-B) functioned properly, even after we verified nominal function of all four devices before flight. The main observable for the non-nominal dosimeters was an apparent loss of sensitivity and dynamic range. We are not aware of any failure mode in the microdosimeter that could replicate the observed states, nor have we seen a similar non-functioning state in many tens of dosimeters flown since the launch of RPS. Figure 15 shows the location of the working dosimeter as well as the response functions for omnidirectional radiation fields.

Being inside the RPS housing meant that we were monitoring the dose behind a significant depth of shielding. The proton response function in Fig. 15 shows a relatively steep turn-on with 10% of the peak response occurring at an incident energy of 53 MeV. This corresponds to a depth of $\sim460$ mils aluminum. The electron response is primarily bremsstrahlung x-rays until direct ionization begins to dominate at about 4.8 MeV. RPS complemented the set of RadFET dose measurements from the ERM sensor (Maurer and Goldsten 2016) and was representative of all the Van Allen Probes subsystems that had a 350 mils box wall requirement.

**Fig. 16:**
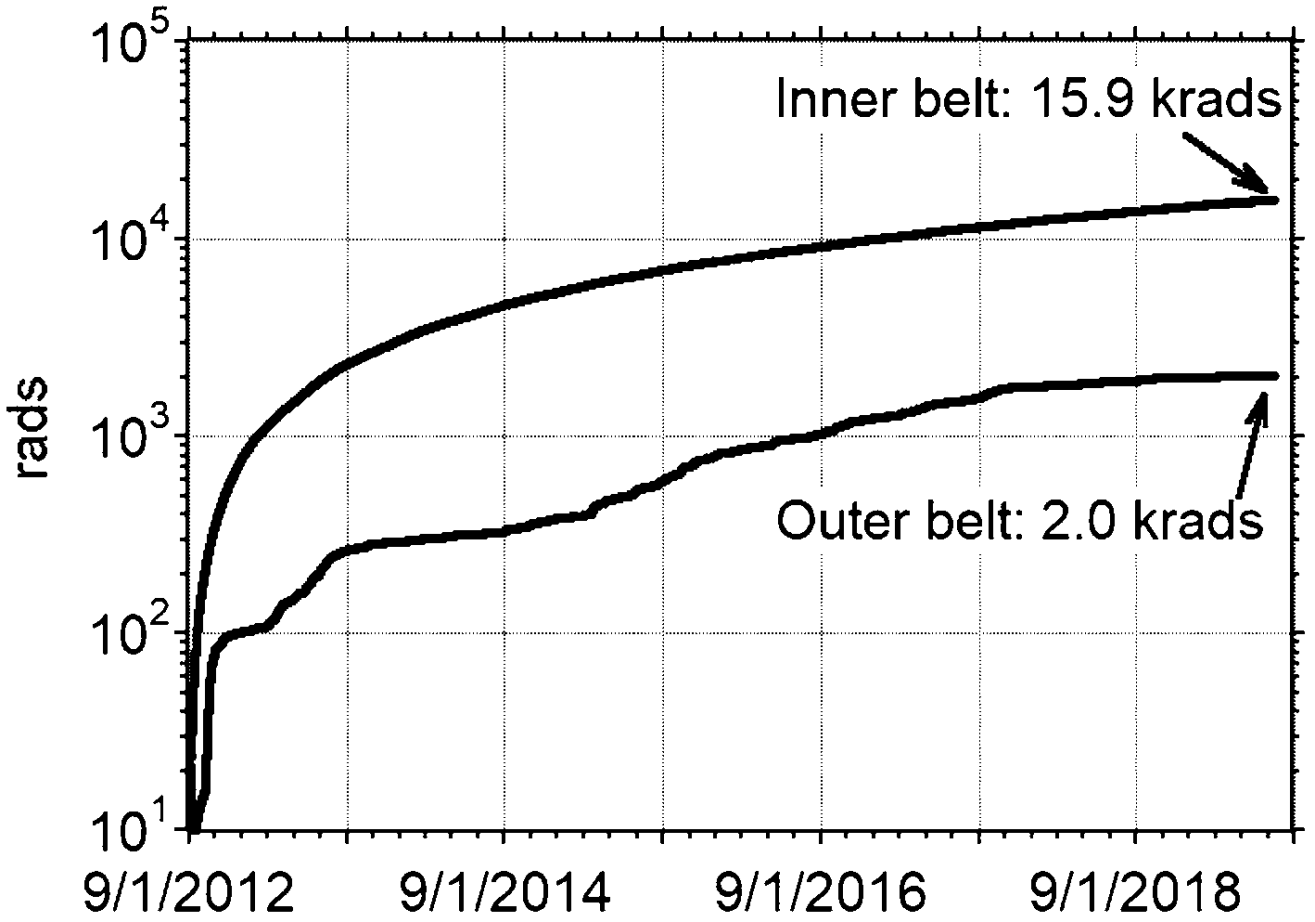
Running accumulations of ionizing dose from RPS-B broken into inner belt ($\text{L}<2.8$) and outer belt ($\text{L}>2.8$). The labels indicate the total mission dose in rads

We were able to separate inner and outer belt contributions to the mission dose because of the dosimeter sensitivity and the once per second dose telemetry. Figure 16 shows that 89% of the total mission dose of 17.9 krads came from the daily inner belt passes, with the rest coming from bremsstrahlung and residual electron contributions from all the outer belt enhancements that we encountered.

**Fig. 17:**
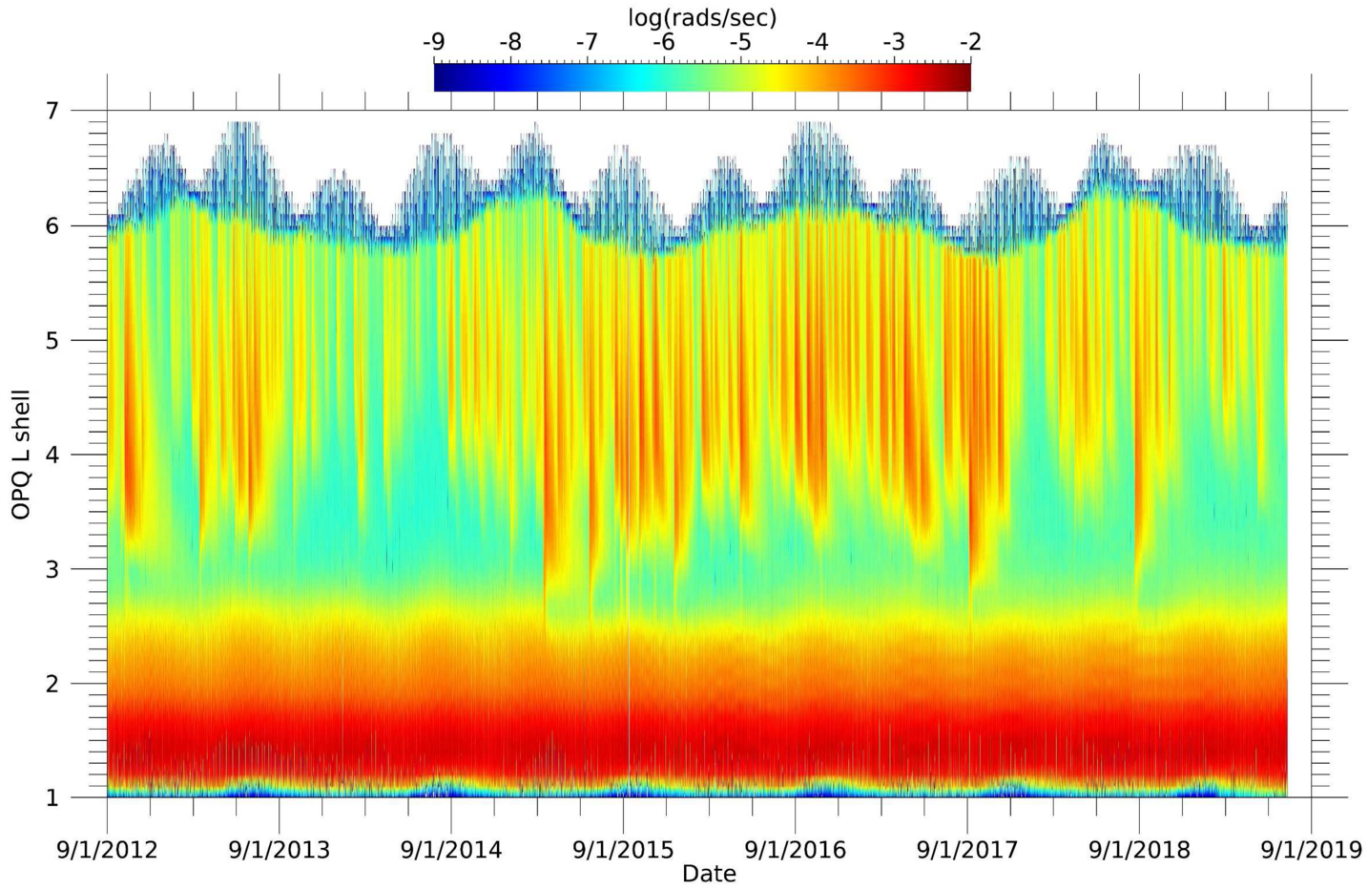
Five-minute averaged dose rate versus L shell from RPS-B Dosimeter 1 for the entire mission

Figure 17 shows the entire mission dataset with dose as a function of L to illustrate more of the time-dependent features. The periodic beating of the swath of high dose in the inner belt arose from orbital sampling of protons nearest the magnetic equator. The intermittent but periodic access of the Van Allen Probes orbit to higher L shells accounted for the features near and above $\text{L}=6$ in the figure. Other interesting features include the step-down in dose near $\text{L}=2.5$ after the 17 March 2015 shock and the enhancements in the dose rate in the slot region at $\text{L}=3$ in several geomagnetic storms where we believe we are measuring x-rays produced by intense soft electrons interacting with RPS and the satellite.

**Fig. 18:**
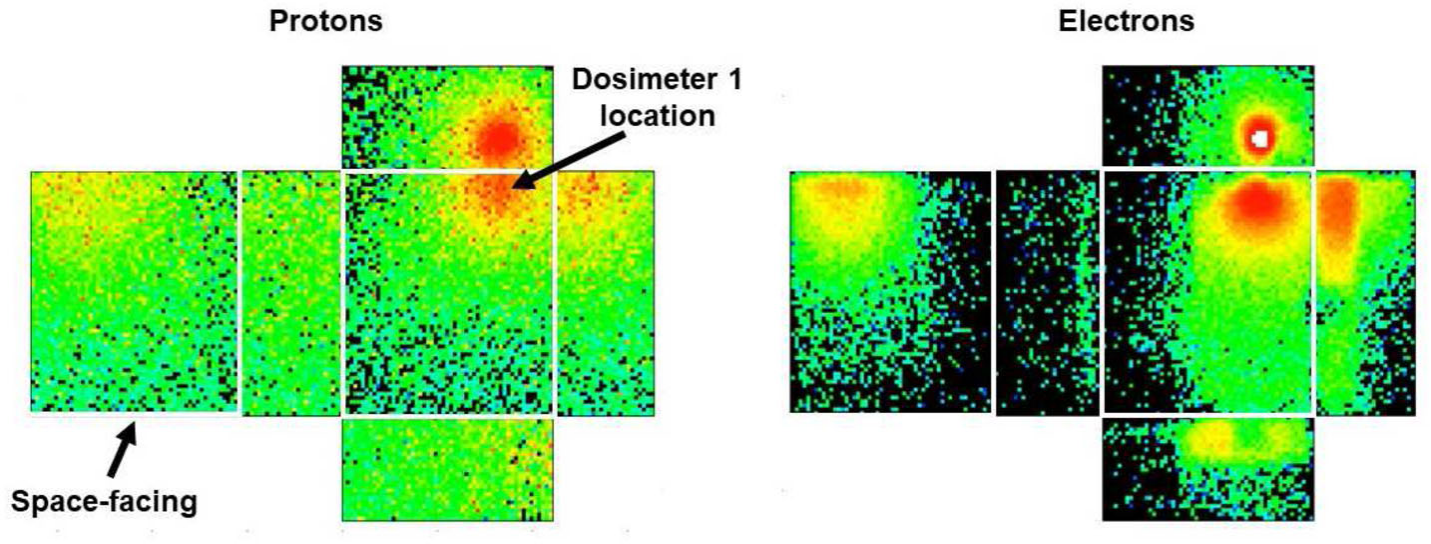
Model of the Dosimeter 1 dose responses projected onto the outer housing of RPS. The color scale (not shown) is logarithmic and results from a convolution of the intrinsic response and representative inner and outer belt energy spectra

For a more detailed study of the angular dependence of dose, we broke out the contributions of the dose from all sides of a RPS as if it was in free space as shown in Fig. 18 using our Geant4 model. We calculated the dose per unit time and incident particle energy by convolving the response functions in Fig. 15 with empirical energy spectra from the Van Allen probes mission. Simulating a free-flying RPS was the first step in seeing how well the model response functions reproduced the measured dose before getting into details of modeling the spacecraft structure.

Since 89% of the dose at any time came from inner belt protons, we focused on the proton response where our model showed that at most one-third of the omnidirectional dose would have been absorbed by the spacecraft. Without detailed modeling of the spacecraft structure, we calculated a 7.3 rads/d rate that was within 9% of the 6.7 rads/d that we measured. That was sufficiently close that we did not pursue modeling of the spacecraft and its mass distribution.

Electrons and their bremsstrahlung radiation dose contributions were not as uniform as the protons because of the effectiveness of the inert shielding around the silicon detector stack, leading to the black areas in the modeled electron response.

Here are some useful orders of magnitude to know for future missions in GTO-like orbits with significant ($>450$ mils) shielding: single passes through the inner belt had peak dose rates of $\sim2$ millirads/s, the worst-case peak outer belt rate was $\sim0.3$ millirads/s, and the daily accumulated dose in quiet time (primarily due to the inner belt) was $\sim6.7$ rads/d.

#### Electrostatic Discharge Counters

The RPS data processing and interface electronics subsystem included on-board monitoring and mitigating of fast ($<120$ nsec width) pulses on the command line and one-pulse-per-second (PPS) timing interfaces to the spacecraft. This was one of the design countermeasures for effects from electrostatic discharges that might have occurred somewhere on the spacecraft (Kirby et al. 2012). The 1-second RPS rate and housekeeping data packet contains these two counters for the entire mission. All counters were reset to zero after an instrument power cycle.

Our routine instrument status and health reporting during the mission indicated that these discharge counters were rarely triggered. Now that all data have been collected, we can summarize what we saw. RPS-A detected a total of five transients on the PPS line (first one on 4/26/2014 and the last three in 2019, the last year of the mission); there were no transients on the RPS-A command line. RPS-B detected a single PPS transient on 10/19/2017 and none on the command line.

We demonstrated our transient detection scheme with ground-based testing and there is no reason to expect that its sensitivity would have changed with time. It is not clear why only the PPS line exhibited these apparent transients. We have not researched the details of the external electron environment at the times of these events or in the lead-up to them. They could have led to sensor anomalies had they not been trapped by the RPS interface system. Future research could interpret them as pseudo-anomalies from electrostatic discharge and perform those comparisons with the instantaneous and time-integrated outer belt electron fluxes.

## Suggestions for Future Missions and Analyses

RPS provides a template for charged particle telescope designs where the target environment has significant penetrating radiation and passive or active shielding are not viable options. Looking beyond the inner proton belt, the compelling evidence for a trapped and stable ultra-relativistic lepton population between $\text{L}\sim 2$ to 3 calls for thinking about alternative sources as well as targeted experimental methods for collecting more information. In this spatial region there exist significant proton fluxes of many hundreds of MeV that are orders of magnitude more intense than the lepton belt. Cherenkov light methods would likely be the best for discriminating against the proton background. Larger geometry factors than RPS would provide better statistics for missions whose orbits quickly pass through this region at high altitude.

Our review of RPS touched on all the science topics that we list here. This is a short summary of what we would suggest for RPS-based investigations going forward, not in priority order.

- Proton fluxes below 500 km after mission perigee-lowering in February and March 2019
- Indirect monitoring of atmospheric scale height using calculated minimum altitude and East-West anisotropy
- Lepton belt: detailed time-history of intensity; pitch angle distribution; full mission statistics of both; alternative source models
- Calibration of RPS detector singles rates for engineering and science to augment Van Allen Probes/ERM dataset
- Detailed study of time dependence and energy dependence of 17 March 2015 shock impact on the outer edge of inner belt
- Detailed intercomparison with other historic inner belt data sets, especially those with digital data records
- Galactic cosmic ray proton inversion.

## Conclusion

At the beginning of the RPS project we described the investigation as one of discovery even though the inner belt is closer to the Earth than most other regions in the magnetosphere and heliosphere. We feel that we achieved a new look at this close yet relatively unexplored region of the magnetosphere. The careful engineering of the Van Allen Probes spacecraft and the dedicated mission operations teams enabled us to close on our primary objective and discover new phenomena along the way.

## Figures and Tables

**Fig. 19:**
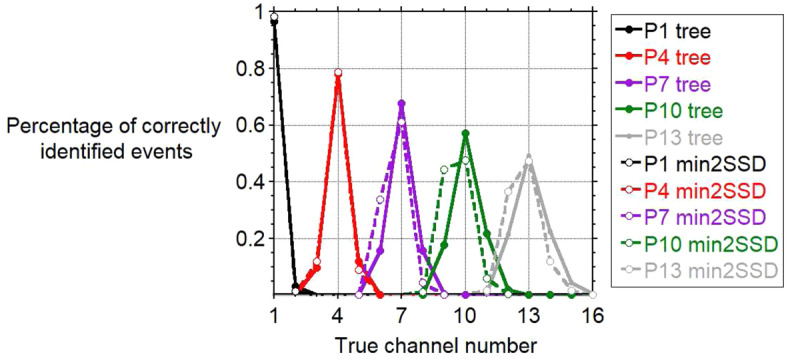
Comparison of the minimum-2 SSD method (dashed lines and open symbols) and decision tree classifier (solid lines and solid symbols) for RPS energy channel assignment. The decision tree classifier was more symmetric than the analytic method, especially at high energies

**Fig. 20:**
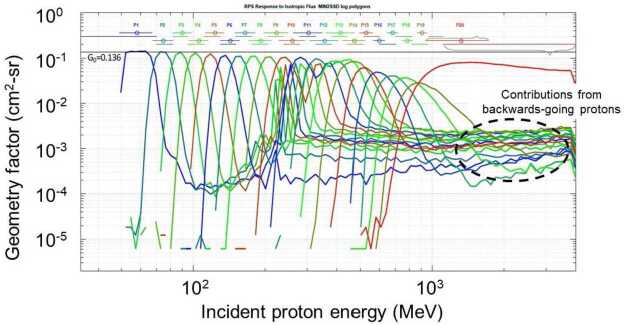
RPS energy channels defined with a bowtie analysis using a falling power-law energy spectrum (O’Brien et al. 2021b)

## Appendix

### A.1 RPS Data Product Levels 0 Through 4

Level 0 data are provided by the mission operations center (MOC) in files containing one full mission day of data in payload telemetry packets (PTP). We convert these packets into NASA CDF format for wider use. PTP files are broken into rate and housekeeping (RH) and direct event (DE) files. Final Level 0 daily filenames follow the pattern rbspa_L0-2c1-rh_psbr-rps_YYYYMMDD_vX.Y.Z.cdf and rbspa_L0-2c2-de_psbr-rps_YYYYMMDD_vX.Y.Z.cdf, where YYYYMMDD is the UTC date most closely matching the mission day.

Level 1 data represent the first real processing of the data where we applied universal time (UTC) tags, calibrations, and coordinates. Level 1 data files merge the rate and housekeeping and direct event data, and contain one full UTC day of data. Level 1 and all higher-level data products are in CDF format. Final Level 1 data files follow the naming pattern rbspa_L1_psbr-rps_YYYYMMDD_vX.Y.Z.cdf, where YYYYMMDD is the UTC date.

Level 2 files are the lowest level that includes particle flux. There are two types of level 2 data files: 1-minute spin averaged files, and sectored files. Level 2 files add magnetic coordinates for multiple magnetic field models. Level 2 sectored files carry forward direct event data in addition to fluxes. Either every minute or every 1/73-spin a flux spectrum is produced in 20 energy channels. A level 2 file contains one UTC day of data. Level 2 sectored files follow the naming pattern rbspa_L2_psbr-rps_YYYYMMDD_vX.Y.Z.cdf, and 1-minute files follow the pattern rbspa_L2-1min_psbr-rps_YYYYMMDD_vX.Y.Z.cdf.

Level 3 data consist of 1-minute accumulations of flux into three different spin subdivisions: spin sectors, local pitch angle bins, and equatorial pitch angle bins. We computed equatorial pitch angle in the Olson-Pfitzer Quiet (OPQ) magnetic field model (Olson and Pfitzer 1977). We calculated a 2-D table of flux versus energy and angle for every minute of the UTC day. Level 3 files follow the naming pattern rbspa_L3_psbr-rps_YYYYMMDD_vX.Y.Z.cdf.

Level 4 data consist of flux maps on three different coordinate systems: E-K-h_min_, E-K-Phi($\Phi $), and E-$\alpha$_eq_-L_m_. E is particle energy, K is the modified second adiabatic invariant (an indicator of motion along the magnetic field direction), hmin is the minimum altitude the particle reaches in its motion around Earth, Phi($\Phi $) is the third adiabatic invariant (amount of magnetic flux enclosed by the particles azimuthal drift around Earth), Alpha_Eq($\alpha$_eq_) is the equatorial pitch angle, and Lm is the McIlwain L value (a radius-like coordinate related to the third invariant). There are two types of level 4 files: daily and by-orbit-leg. For every UTC day or orbit leg we produced a 3-D table of flux in each of these three coordinate systems according to the OPQ magnetic field model. Note that there is a one-to-one mapping between Phi and L*, the third invariant. Level 4 files are computed from level 2 data. Each level 4 file contains one UTC month of data. Level 4 files follow the naming pattern rbspa_L4_psbr-rps_YYYYMMDD_vX.Y.Z.cdf or rbspa_L4-byleg_psbr-rps_YYYYMMDD_vX.Y.Z.cdf, where YYYYMMDD is the UTC date of the first day of data in the file.

### A.2 Post-Launch Calibration

Once we had measurements of the actual space radiation environment with which to compare to our Geant4 model (Mazur et al. 2012), we found several ways in which we needed to modify the simulations in order to better match the response of the real sensors. These changes, in turn, enabled us to improve our ability to use the measurements of energy deposit and Cherenkov light to reject background events and to optimize the determination of proton incident energy.

Mazur et al. (2012) presented a table of the physical processes modeled in the simulations, discussing the relevance of each process to the sensors measurements; Table 4 below is adapted from this, and adds a fourth column to identify the changes that we made to the simulations after launch that have been factored in to the RPS data products.

**Table 4:** Physics processes included in the Geant4 simulation of RPS describing modifications made after launch. Adapted and extended from Table 3 of Mazur et al. (2012) and covered in detail in a report by Looper et al. (2021b)

Physics process	Relevant to these RPS subsystems	Specific model parameters	Changes made to simulation after launch
Geant version	All	V9.2 pre-flight simulation	V10.0 post-flight; QBBC_EMZ reference physics list
Electromagnetic energy loss	SSDA (solid state detector assembly – primary measurement); CRA (Cherenkov radiator assembly – scintillation)	Includes scattering and ionizing energy loss fluctuations for realistic straggling of range and energy deposit	Adjusted SSDA gains to establish consistency between individual detector response to the same proton
Secondary-particle generation	All	Includes creation of knock-on electrons (delta rays) and bremsstrahlung photons that can carry energy away from detector volumes	None
Nuclear interactions	All	Based on Geant4 Binary Cascade model	Used average of two smallest SSDA energy deposits to characterize energy deposit, to filter out randomly occurring large pulse heights that spread out response
Cherenkov radiation	CRA; MCP faceplate	Photon spectral distributions are shaped by wavelength-dependent refractive index of optical materials	None
Scintillation	CRA	Spectral distribution from Viehmann et al. (1975)	Upward adjustments of the yield (photons per unit energy deposit) from preflight estimate
Optical photon transport	CRA; MCP faceplate	Includes wavelength-dependent absorption and partial and total internal reflection	Reduced absorption of black paint on small end of CRA radiator to match observed relative suppression of light from backward protons
Quantum efficiency	MCP photocathode	Wavelength dependence from manufacturer specification	Determined and corrected for variation of overall MCP response

### A.3 Machine Learning Experiments

Although our final mission data products are proton fluxes calculated as described in O’Brien et al. (2021a), we also experimented with machine learning as a method to identify out-of-view protons and to assign events to energy channels. Machine learning has been used in particle physics to identify events of interest for many years using neural networks (e.g., Whiteson and Whiteson 2009), decision trees (e.g., Roe et al. 2005), and other methods (Radovic et al. 2018; and references therein).

For our classification tasks of incident proton energy and out-of-view protons, we used the decision tree classifier provided by the SciKit Learn Python package (scikit-learn.org). A decision tree classifier is a hierarchical binary tree of decisions. At each branch in the tree one of the input parameters for a query case is compared to a threshold, and if it exceeds the threshold, the right branch is taken, otherwise the left branch is taken. Eventually the routine finds the bottom of the tree at a “leaf” node, and it makes a classification based on the category of plurality of training points that also arrived at the same leaf node.

We built the tree structure by choosing the input parameter and best numerical threshold at each decision node to optimize an information gain metric. In simple terms, information gain maximizes how much difference a given split on the data makes on the resulting distribution of categories being classified. For example, when building the classifier that identifies out-of-view particles, it achieves a large information gain from splits that strongly separate in-view protons from out-of-view protons. We used the Geant4 simulation data to train the classifier on the detectable characteristics of in-view or out-of-view protons.

We built the two different classifiers with this general approach. We trained the first classifier to accept D1 through D8 energy deposits as well as D9 photon counts as inputs, with the output being whether the proton was in-view or out-of-view. Geant4 data were partitioned into training and out-of-sample evaluation data. Compared to the polygon-based approach used in our production processing, the decision tree reduced incorrect labelling by about 50% (from $\sim30\%$ to $\sim15\%$) in the 1/E spectrum of the Geant4 simulation. We did not expect the performance advantage to be quite that good for the steeper spectra found in the inner zone, but the result is nonetheless very promising. We would need to ensure that the decision tree is not overfitting on some feature of the simulation that is not reliably present in the real data before we would consider applying this method to the entire RPS dataset.

This in-view versus out-of-view decision tree had a depth of 11, meaning it had on the order of 1000 decision nodes, making it difficult to visualize. The decision tree classifier was comparable in speed to the polygon method, because any individual decision involved only 11 comparisons.

We trained the second classifier to accept D1 through D8 energy deposits (but not D9) as inputs with output being one of the nominal Level-2 channels P1-P20. We omitted D9 to make the comparison equivalent to our MIN2SSD energy assignment algorithm. For this classifier we did not find a significant improvement in the numerical performance measured with the classification success rate. However, we did find that the classifier had more symmetric channel misclassifications than our production algorithm (see Fig. 19). This is a potentially desirable property, even if it does not lead to traditionally superior statistical skill.

As an experiment, we also built a variant of the energy channel classifier that included D9 as an additional input. Adding D9 provided a $\sim30\%$ reduction (out-of-sample) in event channel misidentification in the 1/E energy spectrum of the Geant4 simulation data, with a decision tree of depth of 16. While we do not expect to realize that large an effect in the steeper spectra of the inner zone, it is nonetheless promising. However, as with the out-of-view classifier, we did not implement it in the production processing because we didn’t fully understand how it achieved its results.

Aside from promising quantitative results, this experiment with machine learning illustrated several lessons about the application of decision trees to event classification in energetic particle telescopes. First, decision trees can be built quickly, with a relatively small investment of human labor. Thereafter, they can establish a reasonable benchmark for how well the event classification scheme should ultimately perform, whether generated manually or by machine. This can be a valuable labor-saving benchmark, as it can prevent an endless search for a better classification scheme that does not exist. Second, small decision trees may be easily understood, but the larger decision trees needed for accurate event classification in multi-detector telescopes are not so easy to understand. That leaves a labor-intensive task if one wants to understand and trust the machine-learning algorithm. The primary risk is that the machine may be overfitting features of the simulation which are not fully representative of reality. Finally, we found that complex decision trees execute at runtimes comparable to the tradition polygon and analytical approaches. As long as the users trust the output, their runtime overhead does not stand in the way of this technique being an option for analyzing large data sets

### A.4 Galactic Cosmic Ray Spectrum Inversion

We optimized our routine data processing for a falling inner belt spectrum, roughly power laws with exponents in the range of 2 to 4. Given the RPS energy coverage, we also realized its potential in providing an alternative data source for 1 AU galactic cosmic ray protons.

The GCR proton energy spectrum at 1 AU peaks at about 300 MeV and is relatively flat across the RPS energy range. The GCR environment is also orders of magnitude below the inner belt and those low count rates allow for alternative sensor designs (e.g. anti-coincidence detectors) that are effective at minimizing out-of-view protons. Using anti-coincidence in the inner belt would have led to unacceptable instrument dead-times.

A flat spectrum is not compatible with the bowtie analysis that we used to construct the higher-order data products. Figure 20 shows the bowtie energy channels and their modeled response; one can see the contributions in the channels from backward-going higher-energy protons above $\sim300$ MeV. For a falling spectrum these bowtie definitions reproduce the input spectrum better than 10%.

The GCR environment would have lower peak responses in these channels and a correspondingly higher contribution from backwards-going protons. A multi-channel spectral inversion might work to adequately retrieve the GCR spectrum from RPS.
